# Supplementary material for: Dietary Habits Modify the Association of Physical Exercise with Cognitive Impairment in Community-Dwelling Older Adults
Source: J Clin Med. 2022 Aug 30;11(17):5122. doi: 10.3390/jcm11175122 (PMC9457371; doi:10.3390/jcm11175122)
Supplement: Supplementary file 1 [file jcm-11-05122-s001.zip › jcm-1854122-supplementary.pdf]

Table S1. The associations between physical exercise and prevalent and incident cognitive impairment in total sample and stratified by dietary habits in older adults <80 years.

	No Physical Exercise		Physical Exercise		OR (95% CI) for the Association Between PE and CI Within Each Stratum of Dietary Habits
	N With/Without CI	OR (95% CI)	N With/Without CI	OR (95% CI)	
**Cross-sectional Analyses**					
Total Sample	104/2240	1.0 (reference)	39/1548	1.02 (0.62–1.69)	
				p = 0.933	
Don't Eat Fruits	78/1354	1.0 (reference)	27/712	1.25 (0.72–2.19)	1.30 (0.74–2.28)
				p = 0.431	*p* = 0.370
Eat Fruits	26/886	0.84 (0.46–1.54)	12/836	0.47 (0.18–1.20)	0.51 (0.15–1.77)
		p = 0.575		*p* = 0.113	*p* = 0.291
Don't Eat Vegetables	20/176	1.0 (reference)	11/82	1.37 (0.52–3.58)	0.94 (0.30–2.96)
				p = 0.523	*p* = 0.915
Eat Vegetables	84/2064	0.48 (0.25–0.91)	28/1466	0.45 (0.20–0.98)	0.98 (0.54–1.76)
		p = 0.026		*p* = 0.045	*p* = 0.935
Don't Drink Tea	68/1253	1.0 (reference)	24/801	1.10 (0.58–2.08)	0.98 (0.51–1.91)
				p = 0.779	*p* = 0.964
Drink Tea	36/986	1.34 (0.77–2.33)	15/746	1.25 (0.58–2.68)	0.93 (0.40–2.18)
		p = 0.301		*p* = 0.574	*p* = 0.876
Don't Eat Meat	54/788	1.0 (reference)	16/428	0.89 (0.43–1.82)	0.69 (0.30–1.56)
				p = 0.745	*p* = 0.367
Eat Meat	50/1451	0.65 (0.37–1.15)	23/1119	0.75 (0.38–1.48)	1.12 (0.57–2.22)
		p = 0.140		*p* = 0.406	*p* = 0.739
Don't Eat Fish	70/1354	1.0 (reference)	26/749	1.12 (0.62–2.02)	1.12 (0.60–2.07)
				p = 0.710	*p* = 0.727
Eat Fish	34/885	1.36 (0.75–2.49)	13/798	1.16 (0.50–2.68)	1.01 (0.38–2.67)
		p = 0.310		*p* = 0.729	*p* = 0.985
Don't Eat Eggs	42/795	1.0 (reference)	15/394	1.02 (0.47–2.23)	1.01 (0.44–2.31)
				p = 0.954	*p* = 0.976
Eat Eggs	62/1444	1.01 (0.57–1.78)	24/1153	1.03 (0.51–2.10)	1.04 (0.53–2.03)
		p = 0.972		*p* = 0.933	*p* = 0.912
Don't Eat Food Made from Beans	59/1051	1.0 (reference)	17/545	1.17 (0.59–2.30)	1.01 (0.49–2.10)
				p = 0.658	*p* = 0.973
Eat Food Made from Beans	45/1188	0.95 (0.54–1.64)	22/1002	0.85 (0.41–1.74)	0.97 (0.47–2.03)
		p = 0.842		*p* = 0.655	*p* = 0.945
Don't Eat Salt-preserved Vegetables	68/1235	1.0 (reference)	22/896	1.08 (0.56–2.07)	1.09 (0.55–2.16)
				p = 0.821	*p* = 0.797
Eat Salt-preserved Vegetables	36/1004	1.04 (0.61–1.78)	17/651	0.99 (0.49–2.02)	0.74 (0.32–1.71)
		p = 0.890		*p* = 0.983	*p* = 0.484
Don't Eat Sugar	78/1601	1.0 (reference)	26/1009	1.07 (0.60–1.91)	1.01 (0.55–1.85)
				p = 0.824	*p* = 0.970
Eat Sugar	26/638	1.11 (0.62–2.00)	13/538	1.02 (0.46–2.27)	1.44 (0.50–4.11)

		p = 0.727		p = 0.958	p = 0.499
Don't Eat Garlic	74/1230	1.0 (reference)	19/702	0.82 (0.42–1.58)	0.68 (0.34–1.34)
				p = 0.549	p = 0.266
Eat Garlic	30/1009	0.80 (0.45–1.40)	20/845	1.09 (0.56–2.15)	2.19 (0.93–5.14)
		p = 0.432		p = 0.794	p = 0.072
Don't Eat Milk Products	83/1847	1.0 (reference)	32/951	1.20 (0.70–2.06)	1.15 (0.67–1.97)
				p = 0.496	p = 0.620
Eat Milk Products	21/392	1.20 (0.57–2.53)	7/596	0.57 (0.20–1.62)	0.28 (0.04–2.02)
		p = 0.635		p = 0.290	p = 0.205
Don't Eat Nut Products	101/1923	1.0 (reference)	36/1183	1.04 (0.63–1.73)	1.03 (0.62–1.71)
				p = 0.873	p = 0.921
Eat Nut Products	3/316	0.40 (0.09–1.75)	3/363	0.24 (0.03–1.90)	-
		p = 0.224		p = 0.177	
Don't Eat Mushroom or Algae	98/2021	1.0 (reference)	35/1184	1.02 (0.61–1.70)	0.99 (0.59–1.65)
				p = 0.946	p = 0.959
Eat Mushroom or Algae	6/217	0.71 (0.18–2.83)	4/361	0.78 (0.17–3.62)	-
		p = 0.625		p = 0.746	
Don't Eat Vitamins	98/2118	1.0 (reference)	39/1305	1.12 (0.67–1.85)	1.11 (0.67–1.84)
				p = 0.670	p = 0.697
Eat Vitamins	6/121	1.40 (0.46–4.21)	0/242	-	-
		p = 0.554			
Don't Eat Medicinal Plants	102/2193	1.0 (reference)	38/1432	1.04 (0.63–1.72)	1.03 (0.62–1.70)
				p = 0.870	p = 0.913
Eat Medicinal Plants	2/46	0.71 (0.07–6.93)	1/115	-	-
		p = 0.768			
**Longitudinal Analyses**					
Total Sample	106/1657	1.0 (reference)	44/1132	0.47 (0.28–0.81)	
				p = 0.007	
Don't Eat Fruits	71/995	1.0 (reference)	24/534	0.45 (0.23–0.89)	0.45 (0.21–0.96)
				p = 0.021	p = 0.038
Eat Fruits	35/662	0.72 (0.40–1.27)	20/598	0.37 (0.16–0.84)	0.14 (0.04–0.51)
		p = 0.251		p = 0.018	p = 0.003
Don't Eat Vegetables	10/125	1.0 (reference)	4/57	1.77 (0.26–11.9)	-
				p = 0.557	
Eat Vegetables	96/1532	1.92 (0.60–6.13)	40/1075	0.84 (0.24–2.91)	0.43 (0.24–0.75)
		p = 0.270		p = 0.781	p = 0.003
Don't Drink Tea	57/940	1.0 (reference)	22/599	0.40 (0.18–0.89)	0.26 (0.09–0.74)
				p = 0.024	p = 0.011
Drink Tea	49/716	1.64 (0.92–2.91)	22/532	0.90 (0.42–1.91)	0.40 (0.18–0.92)
		p = 0.092		p = 0.779	p = 0.031
Don't Eat Meat	34/579	1.0 (reference)	12/320	0.48 (0.18–1.31)	0.44 (0.08–2.33)
				p = 0.152	p = 0.338
Eat Meat	72/1077	1.77 (0.94–3.34)	32/811	0.83 (0.38–1.82)	0.46 (0.24–0.89)
		p = 0.079		p = 0.645	p = 0.022

Don’t Eat Fish	73/1007	1.0 (reference)	22/575	0.36 (0.17–0.75)<br>*p* = 0.006	0.36 (0.16–0.84)<br>*p* = 0.018
Eat Fish	33/649	1.15 (0.64–2.07)<br>*p* = 0.630	22/556	0.78 (0.36–1.68)<br>*p* = 0.519	0.48 (0.18–1.24)<br>*p* = 0.130
Don’t Eat Eggs	40/582	1.0 (reference)	14/286	0.36 (0.14–0.93)<br>*p* = 0.034	0.42 (0.12–1.44)<br>*p* = 0.166
Eat Eggs	66/1074	0.76 (0.41–1.39)<br>*p* = 0.368	30/845	0.41 (0.19–0.88)<br>*p* = 0.022	0.49 (0.24–0.99)<br>*p* = 0.046
Don’t Eat Food Made from Beans	50/797	1.0 (reference)	15/410	0.41 (0.18–0.96)<br>*p* = 0.041	0.41 (0.15–1.13)<br>*p* = 0.085
Eat Food Made from Beans	56/859	1.08 (0.61–1.92)<br>*p* = 0.800	29/721	0.56 (0.27–1.18)<br>*p* = 0.126	0.50 (0.23–1.07)<br>*p* = 0.074
Don’t Eat Salt-preserved Vegetables	64/901	1.0 (reference)	23/639	0.38 (0.18–0.80)<br>*p* = 0.011	0.37 (0.15–0.87)<br>*p* = 0.022
Eat Salt-preserved Vegetables	42/755	0.69 (0.40–1.21)<br>*p* = 0.196	21/492	0.42 (0.20–0.89)<br>*p* = 0.023	0.35 (0.14–0.89)<br>*p* = 0.027
Don’t Eat Sugar	62/1203	1.0 (reference)	27/739	0.44 (0.21–0.90)<br>*p* = 0.024	0.31 (0.14–0.68)<br>*p* = 0.004
Eat Sugar	44/453	2.11 (1.20–3.70)<br>*p* = 0.009	17/392	1.10 (0.52–2.33)<br>*p* = 0.811	0.44 (0.14–1.37)<br>*p* = 0.156
Don’t Eat Garlic	58/913	1.0 (reference)	18/524	0.37 (0.15–0.90)<br>*p* = 0.029	0.25 (0.08–0.81)<br>*p* = 0.020
Eat Garlic	48/743	1.54 (0.89–2.68)<br>*p* = 0.125	26/607	0.84 (0.41–1.70)<br>*p* = 0.629	0.69 (0.33–1.41)<br>*p* = 0.307
Don’t Eat Milk Products	91/1371	1.0 (reference)	34/716	0.51 (0.28–0.92)<br>*p* = 0.025	0.53 (0.28–0.98)<br>*p* = 0.043
Eat Milk Products	15/285	0.68 (0.31–1.49)<br>*p* = 0.339	10/415	0.24 (0.08–0.71)<br>*p* = 0.010	-
Don’t Eat Nut Products	93/1404	1.0 (reference)	38/864	0.46 (0.26–0.84)<br>*p* = 0.011	0.44 (0.24–0.82)<br>*p* = 0.009
Eat Nut Products	13/252	0.92 (0.38–2.22)<br>*p* = 0.860	6/266	0.48 (0.16–1.46)<br>*p* = 0.197	-
Don’t Eat Mushroom or Algae	99/1493	1.0 (reference)	39/874	0.49 (0.28–0.85)<br>*p* = 0.011	0.48 (0.28–0.85)<br>*p* = 0.012
Eat Mushroom or Algae	7/163	0.47 (0.15–1.41)<br>*p* = 0.177	5/255	0.16 (0.03–0.78)<br>*p* = 0.023	-
Don’t Eat Vitamins	101/1566	1.0 (reference)	39/966	0.45 (0.26–0.79)<br>*p* = 0.006	0.44 (0.25–0.77)<br>*p* = 0.005
Eat Vitamins	5/90	0.28 (0.05–1.54)<br>*p* = 0.143	5/165	0.28 (0.06–1.33)<br>*p* = 0.110	-
Don’t Eat Medicinal Plants	103/1625	1.0 (reference)	41/1054	0.48 (0.28–0.84)<br>*p* = 0.010	0.48 (0.27–0.84)<br>*p* = 0.010
Eat Medicinal Plants	3/31	3.11 (0.61–15.8)<br>*p* = 0.172	3/77	1.10 (0.22–5.45)<br>*p* = 0.910	-

Table S2. The associations between physical exercise and prevalent and incident cognitive impairment in total sample and stratified by dietary habits in older adults ≥80 years.

	**No Physical Exercise**		**Physical Exercise**		OR (95% CI) for the Association Between PE and CI Within Each Stratum of Dietary Habits
	N With/Without CI	OR (95% CI)	N With/Without CI	OR (95% CI)	
**Cross-sectional Analyses**					
Total Sample	3592/4902	1.0 (reference)	586/1924	0.92 (0.79–1.07) $p = 0.261$	
Don't Eat Fruits	2485/3189	1.0 (reference)	362/1008	1.04 (0.86–1.25) $p = 0.706$	1.03 (0.85–1.24) $p = 0.765$
Eat Fruits	1106/1713	1.07 (0.93–1.24) $p = 0.353$	224/916	0.80 (0.63–1.02) $p = 0.072$	0.76 (0.59–0.98) $p = 0.031$
Don't Eat Vegetables	752/552	1.0 (reference)	72/166	0.80 (0.54–1.19) $p = 0.276$	0.71 (0.48–1.07) $p = 0.100$
Eat Vegetables	2839/4350	0.52 (0.43–0.62) $p<0.001$	514/1758	0.48 (0.39–0.60) $p<0.001$	0.96 (0.81–1.13) $p = 0.589$
Don't Drink Tea	2750/3082	1.0 (reference)	419/1121	0.90 (0.75–1.08) $p = 0.263$	0.94 (0.78–1.13) $p = 0.499$
Drink Tea	840/1820	0.86 (0.75–0.99) $p = 0.037$	165/802	0.82 (0.64–1.05) $p = 0.111$	0.89 (0.68–1.17) $p = 0.407$
Don't Eat Meat	1369/1602	1.0 (reference)	166/524	0.85 (0.65–1.10) $p = 0.211$	0.85 (0.65–1.11) $p = 0.241$
Eat Meat	2221/3300	0.87 (0.75–1.00) $p = 0.054$	419/1399	0.83 (0.68–1.01) $p = 0.067$	0.93 (0.78–1.12) $p = 0.459$
Don't Eat Fish	2463/3074	1.0 (reference)	339/1055	0.89 (0.74–1.08) $p = 0.234$	0.94 (0.77–1.14) $p = 0.505$
Eat Fish	1127/1828	1.06 (0.91–1.23) $p = 0.449$	246/868	1.02 (0.81–1.28) $p = 0.891$	0.88 (0.70–1.12) $p = 0.305$
Don't Eat Eggs	1093/1615	1.0 (reference)	145/488	0.93 (0.71–1.23) $p = 0.610$	0.86 (0.65–1.14) $p = 0.294$
Eat Eggs	2497/3287	1.08 (0.93–1.25) $p = 0.308$	440/1435	0.98 (0.80–1.20) $p = 0.877$	0.93 (0.77–1.11) $p = 0.396$
Don't Eat Food Made from Beans	1781/2230	1.0 (reference)	292/758	1.15 (0.93–1.41) $p = 0.209$	1.12 (0.90–1.40) $p = 0.305$
Eat Food Made from Beans	1809/2671	0.91 (0.79–1.04) $p = 0.153$	293/1165	0.68 (0.55–0.85) $p = 0.001$	0.74 (0.60–0.92) $p = 0.005$
Don't Eat Salt-preserved Vegetables	2645/3168	1.0 (reference)	407/1246	0.88 (0.73–1.05) $p = 0.165$	0.87 (0.72–1.05) $p = 0.145$
Eat Salt-preserved Vegetables	945/1733	0.86 (0.74–0.99) $p = 0.033$	178/677	0.86 (0.67–1.09) $p = 0.209$	0.98 (0.76–1.28) $p = 0.907$
Don't Eat Sugar	2084/3199	1.0 (reference)	309/1176	0.82 (0.68–1.00) $p = 0.050$	0.79 (0.65–0.97) $p = 0.021$

Eat Sugar	1506/1703	1.03 (0.89–1.18)	275/747	1.10 (0.88–1.38)	1.14 (0.89–1.45)
		$p = 0.699$		$p = 0.394$	$p = 0.294$
Don't Eat Garlic	2426/3092	1.0 (reference)	338/1030	0.89 (0.74–1.08)	0.90 (0.74–1.10)
				$p = 0.250$	$p = 0.299$
Eat Garlic	1163/1810	1.08 (0.94–1.24)	247/893	1.03 (0.83–1.29)	0.91 (0.72–1.16)
		$p = 0.259$		$p = 0.774$	$p = 0.462$
Don't Eat Milk Products	2529/3710	1.0 (reference)	375/1136	0.98 (0.82–1.18)	1.00 (0.83–1.19)
				$p = 0.864$	$p = 0.962$
Eat Milk Products	1061/1192	1.02 (0.87–1.20)	210/787	0.81 (0.63–1.04)	0.76 (0.58–1.00)
		$p = 0.785$		$p = 0.103$	$p = 0.052$
Don't Eat Nut Products	3389/4527	1.0 (reference)	543/1684	0.91 (0.78–1.06)	0.91 (0.78–1.07)
				$p = 0.232$	$p = 0.248$
Eat Nut Products	201/375	0.85 (0.65–1.12)	42/239	0.87 (0.55–1.40)	0.94 (0.53–1.68)
		$p = 0.252$		$p = 0.571$	$p = 0.842$
Don't Eat Mushroom or Algae	3317/4460	1.0 (reference)	508/1588	0.93 (0.80–1.09)	0.95 (0.81–1.11)
				$p = 0.386$	$p = 0.523$
Eat Mushroom or Algae	273/442	1.00 (0.79–1.27)	76/335	0.81 (0.55–1.20)	0.67 (0.42–1.07)
		$p = 0.997$		$p = 0.298$	$p = 0.090$
Don't Eat Vitamins	3276/4503	1.0 (reference)	532/1637	0.94 (0.81–1.10)	0.94 (0.80–1.10)
				$p = 0.473$	$p = 0.424$
Eat Vitamins	314/399	1.10 (0.87–1.40)	53/286	0.79 (0.52–1.20)	0.82 (0.49–1.38)
		$p = 0.434$		$p = 0.272$	$p = 0.458$
Don't Eat Medicinal Plants	3505/4765	1.0 (reference)	570/1795	0.92 (0.79–1.07)	0.92 (0.79–1.07)
				$p = 0.275$	$p = 0.296$
Eat Medicinal Plants	85/137	0.54 (0.36–0.83)	15/128	0.48 (0.23–0.99)	0.54 (0.18–1.68)
		$p = 0.004$		$p = 0.048$	$p = 0.289$
**Longitudinal Analyses**					
Total Sample	728/1611	1.0 (reference)	240/806	0.98 (0.74–1.29)	
				$p = 0.862$	
Don't Eat Fruits	480/1045	1.0 (reference)	137/436	0.98 (0.70–1.38)	1.11 (0.78–1.58)
				$p = 0.906$	$p = 0.561$
Eat Fruits	248/566	0.62 (0.45–0.86)	103/370	0.60 (0.38–0.93)	0.86 (0.50–1.49)
		$p = 0.004$		$p = 0.022$	$p = 0.592$
Don't Eat Vegetables	69/167	1.0 (reference)	13/66	0.79 (0.29–2.15)	-
				$p = 0.647$	
Eat Vegetables	659/1444	1.59 (0.95–2.65)	227/740	1.57 (0.90–2.75)	0.97 (0.73–1.31)
		$p = 0.079$		$p = 0.111$	$p = 0.863$
Don't Drink Tea	486/976	1.0 (reference)	163/452	0.93 (0.66–1.32)	0.95 (0.65–1.36)
				$p = 0.688$	$p = 0.763$
Drink Tea	242/635	0.76 (0.55–1.03)	77/354	0.80 (0.52–1.22)	0.94 (0.57–1.56)
		$p = 0.079$		$p = 0.293$	$p = 0.807$
Don't Eat Meat	235/558	1.0 (reference)	44/216	0.84 (0.50–1.41)	0.72 (0.39–1.33)
				$p = 0.505$	$p = 0.296$
Eat Meat	493/1053	1.12 (0.81–1.55)	196/590	1.16 (0.78–1.71)	1.04 (0.74–1.47)

		$p = 0.500$		$p = 0.474$	$p = 0.809$
Don't Eat Fish	468/1041	1.0 (reference)	130/458	0.84 (0.59–1.21)	0.87 (0.59–1.27)
				$p = 0.351$	$p = 0.459$
Eat Fish	260/570	0.93 (0.67–1.28)	110/348	1.13 (0.74–1.72)	1.08 (0.66–1.74)
		$p = 0.649$		$p = 0.585$	$p = 0.768$
Don't Eat Eggs	234/542	1.0 (reference)	67/203	1.17 (0.71–1.92)	1.59 (0.89–2.85)
				$p = 0.533$	$p = 0.118$
Eat Eggs	494/1069	0.87 (0.63–1.21)	173/603	0.79 (0.53–1.18)	0.85 (0.60–1.20)
		$p = 0.410$		$p = 0.245$	$p = 0.356$
Don't Eat Food Made from Beans	326/784	1.0 (reference)	98/329	1.01 (0.67–1.54)	1.23 (0.79–1.93)
				$p = 0.948$	$p = 0.365$
Eat Food Made from Beans	402/826	1.40 (1.04–1.90)	142/477	1.33 (0.89–1.98)	0.93 (0.63–1.36)
		$p = 0.027$		$p = 0.162$	$p = 0.695$
Don't Eat Salt-preserved Vegetables	466/1023	1.0 (reference)	161/522	1.11 (0.79–1.56)	1.01 (0.70–1.44)
				$p = 0.542$	$p = 0.974$
Eat Salt-preserved Vegetables	262/587	1.32 (0.97–1.79)	79/284	1.01 (0.64–1.58)	0.92 (0.54–1.58)
		$p = 0.077$		$p = 0.971$	$p = 0.762$
Don't Eat Sugar	470/1037	1.0 (reference)	134/508	0.85 (0.59–1.22)	0.89 (0.61–1.32)
				$p = 0.371$	$p = 0.577$
Eat Sugar	258/574	1.06 (0.79–1.44)	106/298	1.25 (0.84–1.87)	1.22 (0.77–1.94)
		$p = 0.684$		$p = 0.270$	$p = 0.391$
Don't Eat Garlic	458/1003	1.0 (reference)	125/407	1.06 (0.73–1.54)	1.10 (0.74–1.65)
				$p = 0.754$	$p = 0.630$
Eat Garlic	270/608	1.12 (0.83–1.52)	115/399	0.99 (0.67–1.47)	0.95 (0.60–1.49)
		$p = 0.456$		$p = 0.979$	$p = 0.813$
Don't Eat Milk Products	556/1242	1.0 (reference)	154/471	0.96 (0.68–1.34)	0.98 (0.69–1.40)
				$p = 0.795$	$p = 0.933$
Eat Milk Products	172/369	0.71 (0.50–1.02)	86/335	0.72 (0.47–1.12)	0.82 (0.46–1.43)
		$p = 0.065$		$p = 0.144$	$p = 0.478$
Don't Eat Nut Products	670/1473	1.0 (reference)	222/698	1.01 (0.75–1.35)	1.03 (0.77–1.39)
				$p = 0.968$	$p = 0.829$
Eat Nut Products	58/138	1.23 (0.72–2.11)	18/108	0.91 (0.44–1.87)	-
		$p = 0.449$		$p = 0.788$	
Don't Eat Mushroom or Algae	669/1491	1.0 (reference)	207/657	0.98 (0.73–1.33)	1.03 (0.76–1.40)
				$p = 0.916$	$p = 0.831$
Eat Mushroom or Algae	59/120	0.92 (0.52–1.64)	33/149	0.84 (0.44–1.58)	-
		$p = 0.776$		$p = 0.587$	
Don't Eat Vitamins	672/1500	1.0 (reference)	214/692	0.98 (0.73–1.32)	1.01 (0.75–1.36)
				$p = 0.898$	$p = 0.959$
Eat Vitamins	56/111	1.18 (0.67–2.06)	26/114	1.09 (0.57–2.11)	-
		$p = 0.572$		$p = 0.789$	
Don't Eat Medicinal Plants	711/1580	1.0 (reference)	233/752	1.00 (0.75–1.33)	0.99 (0.74–1.33)
				$p = 0.984$	$p = 0.966$

Eat Medicinal Plants	17/31	1.31 (0.50–3.46) $p = 0.586$	7/54	0.71 (0.25–2.03) $p = 0.529$	-

Table S3. The associations between physical exercise and prevalent and incident cognitive impairment in total sample and stratified by dietary habits in older males.

	No Physical Exercise		Physical Exercise		OR (95% CI) for the Association Between PE and CI Within Each Stratum of Dietary Habits
	N With/Without CI	OR (95% CI)	N With/Without CI	OR (95% CI)	
**Cross-sectional Analyses**					
Total Sample	948/3102	1.0 (reference)	241/1964	0.98 (0.79–1.22) $p = 0.855$	
Don't Eat Fruits	700/2015	1.0 (reference)	152/988	1.10 (0.84–1.44) $p = 0.469$	1.05 (0.80–1.38) $p = 0.726$
Eat Fruits	247/1087	1.06 (0.82–1.37) $p = 0.649$	89/976	0.85 (0.60–1.19) $p = 0.340$	0.85 (0.58–1.25) $p = 0.417$
Don't Eat Vegetables	206/344	1.0 (reference)	35/139	0.91 (0.53–1.58) $p = 0.746$	0.76 (0.42–1.36) $p = 0.354$
Eat Vegetables	741/2758	0.51 (0.38–0.68) $p<0.001$	206/1825	0.50 (0.36–0.70) $p<0.001$	1.04 (0.81–1.32) $p = 0.771$
Don't Drink Tea	644/1616	1.0 (reference)	141/910	0.92 (0.69–1.23) $p = 0.583$	0.95 (0.70–1.27) $p = 0.710$
Drink Tea	304/1485	0.88 (0.70–1.11) $p = 0.280$	100/1053	0.93 (0.68–1.28) $p = 0.659$	1.02 (0.73–1.42) $p = 0.924$
Don't Eat Meat	386/1003	1.0 (reference)	64/505	0.77 (0.52–1.12) $p = 0.175$	0.75 (0.50–1.11) $p = 0.147$
Eat Meat	562/2098	0.81 (0.64–1.03) $p = 0.090$	177/1458	0.89 (0.66–1.21) $p = 0.451$	1.10 (0.84–1.45) $p = 0.468$
Don't Eat Fish	635/1906	1.0 (reference)	131/986	0.97 (0.73–1.29) $p = 0.831$	1.00 (0.75–1.34) $p = 0.991$
Eat Fish	313/1195	1.25 (0.98–1.61) $p = 0.076$	110/977	1.25 (0.90–1.72) $p = 0.182$	0.97 (0.69–1.36) $p = 0.849$
Don't Eat Eggs	314/1073	1.0 (reference)	61/462	1.08 (0.73–1.60) $p = 0.700$	0.92 (0.60–1.39) $p = 0.684$
Eat Eggs	634/2028	1.16 (0.91–1.49) $p = 0.229$	180/1501	1.10 (0.81–1.49) $p = 0.554$	0.97 (0.75–1.26) $p = 0.836$
Don't Eat Food Made from Beans	455/1457	1.0 (reference)	119/710	1.36 (1.00–1.86) $p = 0.049$	1.42 (1.03–1.96) $p = 0.035$
Eat Food Made from Beans	493/1644	1.02 (0.81–1.29) $p = 0.852$	122/1253	0.76 (0.55–1.04) $p = 0.087$	0.72 (0.53–0.98) $p = 0.034$
Don't Eat Salt-preserved Vegetables	675/1921	1.0 (reference)	168/1241	0.98 (0.75–1.27) $p = 0.857$	0.97 (0.74–1.28) $p = 0.835$
Eat Salt-preserved Vegetables	273/1180	1.01 (0.79–1.28) $p = 0.959$	73/722	0.99 (0.71–1.39) $p = 0.969$	0.97 (0.66–1.42) $p = 0.868$

Don't Eat Sugar	637/2128	1.0 (reference)	146/1246	0.94 (0.72–1.23)<br>$p = 0.660$	0.93 (0.71–1.22)<br>$p = 0.592$
Eat Sugar	311/973	0.87 (0.68–1.11)<br>$p = 0.247$	95/717	0.91 (0.65–1.28)<br>$p = 0.601$	1.09 (0.74–1.60)<br>$p = 0.664$
Don't Eat Garlic	628/1834	1.0 (reference)	130/927	0.93 (0.70–1.23)<br>$p = 0.602$	0.92 (0.69–1.23)<br>$p = 0.572$
Eat Garlic	320/1267	0.96 (0.75–1.21)<br>$p = 0.714$	111/1036	1.01 (0.74–1.39)<br>$p = 0.937$	1.02 (0.72–1.44)<br>$p = 0.928$
Don't Eat Milk Products	666/2444	1.0 (reference)	152/1174	1.08 (0.83–1.40)<br>$p = 0.572$	1.11 (0.85–1.44)<br>$p = 0.454$
Eat Milk Products	282/657	1.19 (0.90–1.56)<br>$p = 0.218$	89/789	0.96 (0.67–1.36)<br>$p = 0.806$	0.79 (0.53–1.19)<br>$p = 0.257$
Don't Eat Nut Products	893/2763	1.0 (reference)	225/1623	1.00 (0.79–1.25)<br>$p = 0.974$	0.98 (0.78–1.23)<br>$p = 0.880$
Eat Nut Products	55/338	0.76 (0.48–1.20)<br>$p = 0.242$	16/339	0.60 (0.29–1.21)<br>$p = 0.154$	0.65 (0.22–1.92)<br>$p = 0.438$
Don't Eat Mushroom or Algae	875/2823	1.0 (reference)	212/1573	1.03 (0.81–1.29)<br>$p = 0.831$	1.05 (0.83–1.33)<br>$p = 0.665$
Eat Mushroom or Algae	73/278	1.07 (0.71–1.60)<br>$p = 0.743$	29/389	0.72 (0.41–1.28)<br>$p = 0.266$	0.55 (0.27–1.14)<br>$p = 0.108$
Don't Eat Vitamins	857/2916	1.0 (reference)	223/1688	1.03 (0.82–1.29)<br>$p = 0.827$	1.04 (0.83–1.31)<br>$p = 0.734$
Eat Vitamins	91/185	1.12 (0.73–1.72)<br>$p = 0.605$	18/275	0.68 (0.36–1.30)<br>$p = 0.244$	0.80 (0.30–2.11)<br>$p = 0.657$
Don't Eat Medicinal Plants	916/3034	1.0 (reference)	235/1821	1.02 (0.81–1.27)<br>$p = 0.886$	1.03 (0.82–1.28)<br>$p = 0.823$
Eat Medicinal Plants	32/67	1.04 (0.52–2.09)<br>$p = 0.910$	6/142	0.29 (0.09–1.00)<br>$p = 0.051$	-
**Longitudinal Analyses**					
Total Sample [#]	267/1516	1.0 (reference)	130/1121	0.70 (0.48–1.04)<br>$p = 0.074$	
Don't Eat Fruits	180/952	1.0 (reference)	66/564	0.71 (0.44–1.15)<br>$p = 0.167$	0.84 (0.51–1.39)<br>$p = 0.503$
Eat Fruits	87/564	0.72 (0.44–1.17)<br>$p = 0.185$	64/557	0.49 (0.28–0.88)<br>$p = 0.017$	0.50 (0.23–1.08)<br>$p = 0.078$
Don't Eat Vegetables	28/136	1.0 (reference)	5/69	0.89 (0.19–4.21)<br>$p = 0.885$	-
Eat Vegetables	239/1380	2.93 (1.15–7.51)<br>$p = 0.025$	125/1052	2.04 (0.77–5.41)<br>$p = 0.153$	0.69 (0.47–1.03)<br>$p = 0.073$
Don't Drink Tea	141/758	1.0 (reference)	69/501	0.59 (0.34–1.03)<br>$p = 0.063$	0.69 (0.37–1.29)<br>$p = 0.245$
Drink Tea	126/757	0.86 (0.55–1.35)<br>$p = 0.513$	61/619	0.70 (0.41–1.19)<br>$p = 0.193$	0.76 (0.43–1.34)<br>$p = 0.343$
Don't Eat Meat	84/490	1.0 (reference)	20/290	0.64 (0.31–1.34)	0.54 (0.19–1.52)

				$p = 0.235$	$p = 0.243$
Eat Meat	183/1025	1.26 (0.76–2.08)	110/830	0.91 (0.51–1.63)	0.68 (0.43–1.08)
		$p = 0.372$		$p = 0.761$	$p = 0.106$
Don't Eat Fish	169/926	1.0 (reference)	67/572	0.68 (0.41–1.13)	0.57 (0.32–1.00)
				$p = 0.136$	$p = 0.050$
Eat Fish	98/589	1.34 (0.84–2.14)	63/548	0.99 (0.57–1.71)	0.69 (0.36–1.31)
		$p = 0.218$		$p = 0.959$	$p = 0.259$
Don't Eat Eggs	83/528	1.0 (reference)	28/256	1.00 (0.50–1.98)	1.13 (0.44–2.89)
				$p = 0.992$	$p = 0.796$
Eat Eggs	184/987	1.06 (0.65–1.73)	102/864	0.65 (0.37–1.15)	0.60 (0.37–0.96)
		$p = 0.820$		$p = 0.140$	$p = 0.034$
Don't Eat Food Made from Beans	124/737	1.0 (reference)	52/417	0.73 (0.42–1.28)<br>$p = 0.274$	0.83 (0.44–1.55)<br>$p = 0.553$
Eat Food Made from Beans	143/778	1.12 (0.72–1.76)<br>$p = 0.611$	78/703	0.77 (0.44–1.34)<br>$p = 0.354$	0.69 (0.40–1.19)<br>$p = 0.179$
Don't Eat Salt-preserved Vegetables	165/905	1.0 (reference)	91/685	0.92 (0.57–1.47)<br>$p = 0.726$	0.84 (0.51–1.40)<br>$p = 0.510$
Eat Salt-preserved Vegetables	102/610	1.34 (0.85–2.12)<br>$p = 0.205$	39/435	0.60 (0.33–1.09)<br>$p = 0.095$	0.46 (0.22–0.99)<br>$p = 0.047$
Don't Eat Sugar	175/1037	1.0 (reference)	75/762	0.55 (0.33–0.91)	0.54 (0.31–0.92)
				$p = 0.020$	$p = 0.024$
Eat Sugar	92/478	0.95 (0.60–1.52)	55/394	0.94 (0.56–1.60)	1.40 (0.70–2.80)
		$p = 0.844$		$p = 0.832$	$p = 0.345$
Don't Eat Garlic	168/867	1.0 (reference)	60/516	0.63 (0.37–1.07)	0.53 (0.29–0.98)
				$p = 0.087$	$p = 0.041$
Eat Garlic	99/648	0.98 (0.63–1.55)	70/604	0.78 (0.47–1.30)	0.74 (0.39–1.38)
		$p = 0.948$		$p = 0.337$	$p = 0.339$
Don't Eat Milk Products	196/1246	1.0 (reference)	81/685	0.80 (0.51–1.27)<br>$p = 0.348$	0.75 (0.46–1.23)<br>$p = 0.256$
Eat Milk Products	71/269	1.24 (0.73–2.12)<br>$p = 0.426$	49/435	0.68 (0.38–1.23)<br>$p = 0.202$	0.27 (0.16–0.63)<br>$p = 0.002$
Don't Eat Nut Products	242/1307	1.0 (reference)	119/903	0.77 (0.51–1.16)<br>$p = 0.210$	0.84 (0.55–1.27)<br>$p = 0.407$
Eat Nut Products	25/208	1.34 (0.67–2.67)<br>$p = 0.410$	11/216	0.49 (0.20–1.21)<br>$p = 0.123$	-
Don't Eat Mushroom or Algae	238/1396	1.0 (reference)	109/895	0.70 (0.47–1.06)<br>$p = 0.090$	0.67 (0.44–1.03)<br>$p = 0.066$
Eat Mushroom or Algae	29/119	1.07 (0.47–2.42)<br>$p = 0.875$	21/224	0.77 (0.36–1.66)<br>$p = 0.504$	-
Don't Eat Vitamins	248/1448	1.0 (reference)	116/979	0.71 (0.48–1.07)<br>$p = 0.100$	0.68 (0.45–1.03)<br>$p = 0.068$
Eat Vitamins	19/67	1.26 (0.49–3.25)<br>$p = 0.630$	14/141	0.77 (0.32–1.83)<br>$p = 0.547$	-

Don't Eat Medicinal Plants	259/1490	1.0 (reference)	126/1042	0.74 (0.50–1.10) $p = 0.135$	0.73 (0.49–1.08) $p = 0.113$
Eat Medicinal Plants	8/25	2.07 (0.53–8.13) $p = 0.296$	4/78	0.42 (0.11–1.59) $p = 0.200$	-

Table S4. The associations between physical exercise and prevalent and incident cognitive impairment in total sample and stratified by dietary habits in older females.

	**No Physical Exercise**		**Physical Exercise**		OR (95% CI) for the Association Between PE and CI Within Each Stratum of Dietary Habits
	N With/Without CI	OR (95% CI)	N With/Without CI	OR (95% CI)	
**Cross-sectional Analyses**					
Total Sample	2748/4040	1.0 (reference)	384/1508	0.87 (0.72–1.05) $p = 0.150$	
Don't Eat Fruits	1863/2528	1.0 (reference)	237/732	1.02 (0.81–1.29) $p = 0.869$	1.02 (0.80–1.29) $p = 0.899$
Eat Fruits	885/1512	1.06 (0.89–1.26) $p = 0.507$	147/776	0.70 (0.51–0.96) $p = 0.027$	0.66 (0.48–0.93) $p = 0.016$
Don't Eat Vegetables	566/384	1.0 (reference)	48/109	0.85 (0.52–1.40) $p = 0.525$	0.80 (0.49–1.32) $p = 0.382$
Eat Vegetables	2182/3656	0.51 (0.41–0.63) $p<0.001$	336/1399	0.44 (0.34–0.58) $p<0.001$	0.89 (0.72–1.09) $p = 0.255$
Don't Drink Tea	2174/2719	1.0 (reference)	302/1012	0.91 (0.73–1.13) $p = 0.388$	0.94 (0.76–1.18) $p = 0.610$
Drink Tea	572/1321	0.88 (0.75–1.05) $p = 0.162$	80/495	0.67 (0.47–0.97) $p = 0.034$	0.72 (0.49–1.07) $p = 0.103$
Don't Eat Meat	1037/1387	1.0 (reference)	118/447	0.90 (0.66–1.25) $p = 0.540$	0.91 (0.66–1.26) $p = 0.578$
Eat Meat	1709/2653	0.86 (0.72–1.02) $p = 0.082$	265/1060	0.73 (0.57–0.95) $p = 0.017$	0.83 (0.65–1.05) $p = 0.121$
Don't Eat Fish	1898/2522	1.0 (reference)	234/818	0.88 (0.70–1.12) $p = 0.312$	0.90 (0.71–1.15) $p = 0.412$
Eat Fish	848/1518	1.02 (0.86–1.22) $p = 0.818$	149/689	0.86 (0.63–1.17) $p = 0.344$	0.81 (0.59–1.10) $p = 0.176$
Don't Eat Eggs	821/1337	1.0 (reference)	99/420	0.88 (0.62–1.24) $p = 0.464$	0.83 (0.58–1.18) $p = 0.289$
Eat Eggs	1925/2703	1.05 (0.88–1.25) $p = 0.594$	284/1087	0.91 (0.70–1.17) $p = 0.452$	0.88 (0.70–1.11) $p = 0.265$
Don't Eat Food Made from Beans	1385/1824	1.0 (reference)	190/593	0.99 (0.76–1.30) $p = 0.947$	0.93 (0.70–1.23) $p = 0.602$
Eat Food Made from Beans	1361/2215	0.86 (0.73–1.01) $p = 0.070$	193/914	0.66 (0.51–0.87) $p = 0.004$	0.79 (0.61–1.04) $p = 0.094$
Don't Eat Salt-preserved	2038/2482	1.0 (reference)	261/901	0.82 (0.65–1.03) $p = 0.094$	0.81 (0.64–1.02) $p = 0.072$

Vegetables					
Eat Salt-preserved Vegetables	708/1557	0.80 (0.68–0.95) $p = 0.010$	122/606	0.78 (0.57–1.07) $p = 0.119$	1.02 (0.73–1.42) $p = 0.916$
Don't Eat Sugar	1525/2672	1.0 (reference)	189/939	0.74 (0.57–0.95) $p = 0.018$	0.72 (0.56–0.93) $p = 0.012$
Eat Sugar	1221/1368	1.14 (0.97–1.35) $p = 0.110$	193/568	1.23 (0.93–1.63) $p = 0.142$	1.11 (0.82–1.49) $p = 0.507$
Don't Eat Garlic	1872/2488	1.0 (reference)	227/805	0.84 (0.66–1.07) $p = 0.154$	0.84 (0.66–1.08) $p = 0.180$
Eat Garlic	873/1552	1.12 (0.95–1.33) $p = 0.164$	156/702	1.03 (0.77–1.38) $p = 0.821$	0.89 (0.65–1.21) $p = 0.457$
Don't Eat Milk Products	1946/3113	1.0 (reference)	255/913	0.95 (0.76–1.19) $p = 0.673$	0.94 (0.75–1.18) $p = 0.591$
Eat Milk Products	800/927	0.97 (0.80–1.17) $p = 0.767$	128/594	0.69 (0.49–0.96) $p = 0.027$	0.69 (0.48–1.01) $p = 0.054$
Don't Eat Nut Products	2597/3687	1.0 (reference)	354/1244	0.85 (0.70–1.04) $p = 0.115$	0.86 (0.70–1.05) $p = 0.130$
Eat Nut Products	149/353	0.82 (0.59–1.14) $p = 0.233$	29/263	0.90 (0.50–1.62) $p = 0.715$	1.00 (0.46–2.17) $p = 0.996$
Don't Eat Mushroom or Algae	2540/3658	1.0 (reference)	331/1199	0.87 (0.71–1.06) $p = 0.176$	0.87 (0.71–1.07) $p = 0.180$
Eat Mushroom or Algae	206/381	1.00 (0.75–1.34) $p = 0.990$	51/307	0.87 (0.52–1.45) $p = 0.584$	0.88 (0.46–1.68) $p = 0.698$
Don't Eat Vitamins	2517/3705	1.0 (reference)	348/1254	0.89 (0.73–1.09) $p = 0.264$	0.88 (0.72–1.07) $p = 0.194$
Eat Vitamins	229/335	1.12 (0.84–1.48) $p = 0.445$	35/253	0.78 (0.46–1.34) $p = 0.372$	0.84 (0.43–1.64) $p = 0.603$
Don't Eat Medicinal Plants	2691/3924	1.0 (reference)	373/1406	0.86 (0.70–1.04) $p = 0.114$	0.85 (0.70–1.03) $p = 0.106$
Eat Medicinal Plants	55/116	0.40 (0.24–0.67) $p = 0.001$	10/101	0.58 (0.23–1.45) $p = 0.246$	1.26 (0.12–13.3) $p = 0.847$
**Longitudinal Analyses**					
Total Sample	567/1752	1.0 (reference)	154/817	0.89 (0.64–1.24) $p = 0.501$	
Don't Eat Fruits	371/1088	1.0 (reference)	95/406	0.92 (0.62–1.38) $p = 0.696$	0.99 (0.65–1.49) $p = 0.945$
Eat Fruits	196/664	0.64 (0.45–0.91) $p = 0.014$	59/411	0.54 (0.32–0.92) $p = 0.023$	0.67 (0.36–1.23) $p = 0.194$
Don't Eat Vegetables	51/156	1.0 (reference)	12/54	1.01 (0.32–3.15) $p = 0.987$	-
Eat Vegetables	516/1596	1.47 (0.85–2.54) $p = 0.167$	142/763	1.30 (0.71–2.39) $p = 0.397$	0.87 (0.62–1.23) $p = 0.432$
Don't Drink Tea	402/1158	1.0 (reference)	116/550	0.91 (0.61–1.34)	0.86 (0.57–1.31)

				$p = 0.617$	$p = 0.489$
Drink Tea	165/594	0.97 (0.69–1.37)	38/267	0.84 (0.49–1.45)	0.91 (0.48–1.74)
		$p = 0.859$		$p = 0.531$	$p = 0.779$
Don't Eat Meat	185/647	1.0 (reference)	36/246	0.90 (0.51–1.60)	0.72 (0.36–1.49)
				$p = 0.719$	$p = 0.355$
Eat Meat	382/1105	1.34 (0.94–1.92)	118/571	1.19 (0.76–1.88)	0.90 (0.60–1.35)
		$p = 0.104$		$p = 0.444$	$p = 0.614$
Don't Eat Fish	372/1122	1.0 (reference)	85/461	0.76 (0.51–1.14)	0.82 (0.53–1.27)
				$p = 0.187$	$p = 0.375$
Eat Fish	195/630	0.80 (0.56–1.15)	69/356	0.96 (0.58–1.61)	0.94 (0.52–1.72)
		$p = 0.235$		$p = 0.881$	$p = 0.851$
Don't Eat Eggs	191/596	1.0 (reference)	53/233	0.85 (0.48–1.49)	1.24 (0.65–2.38)
				$p = 0.559$	$p = 0.511$
Eat Eggs	376/1156	0.78 (0.55–1.12)	101/584	0.72 (0.45–1.14)	0.94 (0.62–1.42)
		$p = 0.179$		$p = 0.160$	$p = 0.767$
Don't Eat Food Made from Beans	252/844	1.0 (reference)	61/322	0.91 (0.55–1.49) $p = 0.696$	0.95 (0.55–1.63) $p = 0.840$
Eat Food Made from Beans	315/907	1.50 (1.07–2.09) $p = 0.018$	93/495	1.32 (0.84–2.10) $p = 0.230$	0.86 (0.55–1.35) $p = 0.514$
Don't Eat Salt-preserved Vegetables	365/1019	1.0 (reference)	93/476	0.86 (0.57–1.30) $p = 0.476$	0.82 (0.53–1.26) $p = 0.358$
Eat Salt-preserved Vegetables	202/732	1.03 (0.74–1.45) $p = 0.842$	61/341	0.98 (0.59–1.61) $p = 0.934$	0.94 (0.53–1.65) $p = 0.820$
Don't Eat Sugar	357/1203	1.0 (reference)	86/521	0.85 (0.56–1.31)	0.85 (0.54–1.34)
				$p = 0.472$	$p = 0.478$
Eat Sugar	210/549	1.39 (1.00–1.93)	68/296	1.31 (0.82–2.11)	1.01 (0.59–1.72)
		$p = 0.052$		$p = 0.260$	$p = 0.972$
Don't Eat Garlic	348/1049	1.0 (reference)	83/415	1.14 (0.73–1.77)	1.18 (0.73–1.90)
				$p = 0.565$	$p = 0.492$
Eat Garlic	219/703	1.45 (1.04–2.02)	71/402	1.00 (0.63–1.59)	0.72 (0.43–1.20)
		$p = 0.027$		$p = 0.991$	$p = 0.206$
Don't Eat Milk Products	451/1367	1.0 (reference)	107/502	0.83 (0.57–1.23) $p = 0.358$	0.85 (0.57–1.27) $p = 0.439$
Eat Milk Products	116/385	0.54 (0.36–0.83) $p = 0.005$	47/315	0.58 (0.33–0.99) $p = 0.047$	0.72 (0.32–1.58) $p = 0.408$
Don't Eat Nut Products	521/1570	1.0 (reference)	141/659	0.89 (0.63–1.25) $p = 0.497$	0.86 (0.60–1.22) $p = 0.385$
Eat Nut Products	46/182	1.01 (0.54–1.87) $p = 0.987$	13/158	0.95 (0.43–2.11) $p = 0.902$	-
Don't Eat Mushroom or Algae	530/1588	1.0 (reference)	137/636	0.93 (0.66–1.31) $p = 0.682$	0.94 (0.67–1.33) $p = 0.741$
Eat Mushroom or	37/164	0.67 (0.35–1.28)	17/180	0.40 (0.16–1.00)	-

Algae		*p* = 0.224		*p* = 0.051	
Don’t Eat Vitamins	525/1618	1.0 (reference)	137/679	0.89 (0.63–1.25) *p* = 0.489	0.89 (0.63–1.27) *p* = 0.533
Eat Vitamins	42/134	0.89 (0.48–1.65) *p* = 0.711	17/138	0.86 (0.38–1.95) *p* = 0.713	-
Don’t Eat Medicinal Plants	555/1715	1.0 (reference)	148/764	0.89 (0.64–1.25) *p* = 0.507	0.90 (0.64–1.26) *p* = 0.530
Eat Medicinal Plants	12/37	1.49 (0.51–4.34) *p* = 0.461	6/53	1.35 (0.40–4.53) *p* = 0.623	-
